# Comparison of Achalasia Classification Schemes to Predict Treatment Outcomes

**DOI:** 10.1111/nmo.70249

**Published:** 2026-01-20

**Authors:** Dustin A. Carlson, Eric Goudie, Jacob M. Schauer, Domenico A. Farina, Leya Chambo, Linda Kelahan, John E. Pandolfino

**Affiliations:** ^1^ Division of Gastroenterology and Hepatology, Department of Medicine, Feinberg School of Medicine, Kenneth C. Griffin Esophageal Center of Northwestern Medicine Northwestern University Chicago Illinois USA; ^2^ Division of Thoracic Surgery, Department of Surgery Université de Montréal Montreal Quebec Canada; ^3^ Division of Biostatistics and Informatics, Department of Preventive Medicine, Feinberg School of Medicine Northwestern University Chicago Illinois USA; ^4^ Department of Radiology, Feinberg School of Medicine Northwestern University Chicago Illinois USA

**Keywords:** dysphagia, manometry, myotomy, outcomes

## Abstract

**Background and Aims:**

Achalasia classifications, such as the Chicago Classification subtypes based on high‐resolution manometry (HRM) and Japanese Esophageal Society (JES), Italian, or Brazilian classifications based on esophagram, have been described. We aimed to compare these schemes for prediction of achalasia treatment outcomes.

**Methods:**

222 adult patients with achalasia that completed pretreatment HRM and esophagram before and after treatment were included. Pretreatment HRM achalasia subtypes were determined by the Chicago Classification and JES; Italian and Brazilian classifications were defined by pretreatment esophagram. Post‐treatment outcomes were defined using the Eckardt symptom score (good outcome < 4) or timed barium esophagram (TBE; good outcome 5‐min column height < 5 cm).

**Results:**

The Chicago Classification was a significant predictor of symptomatic outcome (*p* = 0.003–0.007), whereas JES, Italian, and Brazilian schemes were not. All four classifications were significant predictors of radiographic outcome, with JES demonstrating the best model fit as identified by lowest Akaike information criteria (AIC). Type III achalasia (Chicago Classification) patients had the lowest rates of good symptomatic outcomes despite treatment primarily with POEM, whereas advanced esophagram stages (JES‐C, Italian IV, Brazilian 2–3) were associated with poorer radiographic outcomes.

**Conclusions:**

Both HRM‐ and esophagram‐based classification schemes predict achalasia treatment outcomes, though with different strengths. While treatment choice may impact outcomes, HRM best predicted symptomatic outcomes, while esophagram classifications better predicted objective radiographic outcomes. Utilizing both modalities may enhance prognostication of outcomes in achalasia.

## KEYPOINTS

- Achalasia classification schemes using high‐resolution manometry (Chicago Classification) or barium esophagram (Japanese, Italian, or Brazilian classification schemes) has been described and may offer prognostic information regarding future treatment outcomes.
- The Chicago Classification based on pressurization and contractility patterns on high‐resolution manometry more strongly predicted symptom‐based treatment outcomes than esophagram‐based schemes, while the esophagram‐based schemes based on esophageal anatomy were stronger predictors for objective measures of esophageal retention.
- Complementary evaluation in achalasia using tools to assess esophageal physiology (such as high‐resolution manometry) and anatomy (such as esophagram) may leverage strengths for predicting treatment responses in achalasia.

## Graphical abstract

Achalasia classification methods can be beneficial to help prognosticate treatment outcomes. High‐resolution manometry subtypes (Chicago Classification) predicted symptomatic outcome, whereas esophagram‐based classifications (Japanese Esophageal Society (JES), Italian, Brazilian) more strongly predicted objective emptying. This highlights the potential complementary value of dual‐modality assessment.

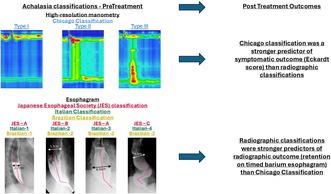

**Abbreviations**
AIC: Akaike information criteria
AUROC: area under the receiver operating characteristic curve
CI: confidence interval
EGJ: esophagogastric junction
GLM: generalized linear models
HRM: high‐resolution manometry
IRP: integrated relaxation pressure
JES: Japanese Esophageal Society
LES: lower esophageal sphincter
LHM: laparoscopic Heller's myotomy
POEM: PerOral Endoscopic Myotomy
TBE: timed barium esophagram

## 1 Introduction

Approaches to classify achalasia are appealing to inform prognosis and help guide treatment decisions. A novel classification for achalasia based on high‐resolution manometry (HRM) patterns was described in 2008 and has been represented in The Chicago Classification for diagnosis of esophageal motility disorders across its multiple updates [1, 2]. All subtypes were characterized by an elevated integrated relaxation pressure (IRP) and absent peristalsis, with type I (classic) further characterized by minimal esophageal pressurization, type II by the presence of panesophageal pressurization, and type III (spastic) by the presence of premature contractions.

The clinical relevance of the classification was demonstrated based on prognostication of treatment response in the initial study, with multiple subsequent studies from other centers verifying and extending these findings [1, 3, 4, 5, 6, 7]. They showed that type II achalasia had the best symptomatic outcome and type III achalasia patients had the worst symptomatic outcome, with type I achalasia exhibiting intermediate outcomes after achalasia treatment. Further, post hoc analysis of the European Achalasia Trial (a randomized clinical trial of pneumatic dilation vs. laparoscopic Heller's myotomy (LHM)) demonstrated that although the treatment outcome of type III achalasia was significantly worse when treated with pneumatic dilation, treatment outcomes were similar to type I and II when treated with LHM [5]. Hence, surgical myotomy has become the recommended treatment approach in type III achalasia [8].

Further, the achalasia landscape has evolved with introduction and widespread adoption of PerOral Endoscopic Myotomy (POEM) as a safe and effective treatment option [9, 10]. POEM has also facilitated tailoring myotomy, either making an extended myotomy to ablate spasm in “type III” achalasia or potentially doing only a short myotomy focused on the LES high pressure zone in non‐spastic subtypes (I–II) [11]. However, subsequent randomized studies with POEM (vs. LHM and vs. pneumatic dilation) have not demonstrated a significant interaction between achalasia HRM subtype and treatment type and outcomes.

Other achalasia classifications focused entirely on esophageal anatomy assessed radiographically were also described by the Japanese Esophageal Society (JES), an Italian group, and a Brazilian group [1, 12, 13]. These intended to reflect the chronic remodeling process observed in achalasia that involves esophageal dilatation and deformity. The outcome prediction with the specific esophagram classifications is less thoroughly described or validated in external cohorts than with the Chicago Classification [1, 3, 4, 5, 6, 7, 14]. Further, no previous study has directly compared these four schemes relative to their abilities to predict treatment outcomes in achalasia. Hence, this study aimed to examine and compare the Chicago Classification HRM subtypes and esophagram (JES, Italian, and Brazilian) classifications for prediction of treatment outcomes in a retrospective cohort study of achalasia.

## 2 Methods

### 2.1 Subjects

This observational cohort study included consecutive patients with treatment‐naïve, idiopathic achalasia (HRM Chicago Classification subtypes I, II, or III) diagnosed from 2014 to 2023 who completed evaluation with baseline (pre‐treatment) HRM, pre‐treatment esophagram, and follow‐up esophagram after treatment with pneumatic dilation, POEM, or LHM. Patients meeting these criteria were retrospectively identified for inclusion from an esophageal motility registry. The registry is prospectively maintained and includes adult patients (ages 18–89 years) who present to the Esophageal Center of Northwestern Medicine for evaluation of esophageal symptoms and management of esophageal conditions. Patients with a history of foregut surgery (including POEM) or pneumatic dilation prior to baseline HRM were excluded, as were patients without available pre or post‐treatment esophagram (Figure S1).

Treatment for achalasia was performed per our center's standard of care with treatment modality determined by shared decision making between patients and their treating provider. Our standard practice is to offer pneumatic dilation, POEM, or LHM to patients with type I or type II achalasia and to preferentially treat patients with type III achalasia with POEM (myotomy length tailored by extent of spasm on HRM). However, insurance coverage and/or patient preference sometimes dictated other treatments. All therapeutic procedures were completed at our center by experienced providers with expertise in achalasia management. While esophagrams were reviewed and anatomy considered during treatment planning, the JES, Italian, or Brazilian classifications were not routinely assessed or applied. Patients treated solely with LES botulinum toxin injection were not included as this was typically offered only to patients with significant comorbidities or diagnostic uncertainty, which would bias assessing treatment outcomes.

Our standard of care follow‐up after achalasia treatment was evaluation including timed barium esophagram (TBE) and Eckardt symptom scores 6–12 months after treatment, or earlier if symptoms persist or recurred [15]. Patients who completed an initial treatment follow‐up evaluation outside of this time window were also included, with actual time to follow‐up assessed. Patients who did not complete the follow‐up Eckardt score, or who had an esophagram performed without a TBE protocol, were still included in the study. The study protocol was approved by the Northwestern University Institutional Review Board as minimal risk with a waiver of informed consent for retrospective analysis of deidentified patient data.

### 2.2 HRM Protocol and Analysis

HRM studies were completed after a 6‐h fast using a 4.2‐mm outer diameter solid‐state assembly with 36 circumferential pressure sensors at 1‐cm intervals (Medtronic, Shoreview, MN). The HRM assembly was placed transnasally and positioned to record from the hypopharynx to the stomach with approximately 3 intragastric sensors. After a 2‐min baseline recording, the HRM protocol was performed including 10, 5‐mL liquid swallows in a supine position and then five, 5‐mL liquid swallows in an upright position [4]. Studies were analyzed using commercially available software (ManoView, Medtronic) according to the Chicago Classification [2, 16, 17].

### 2.3 Barium Esophagram Protocol, Analysis, and Outcomes

Barium esophagrams were performed in the upright position. For TBEs, patients consumed 200 mL of low‐density barium sulfate with anteroposterior images obtained at 1, 2, and 5 min [18]. Esophagrams at baseline and follow‐up were reviewed by study team members (EG, DF, LK), who were blinded to clinical characteristics, including HRM findings and treatment details. Esophagram analyses were performed using Visage software (San Diego, CA, USA) available through the Northwestern electronic medical record.

**FIGURE 1:**
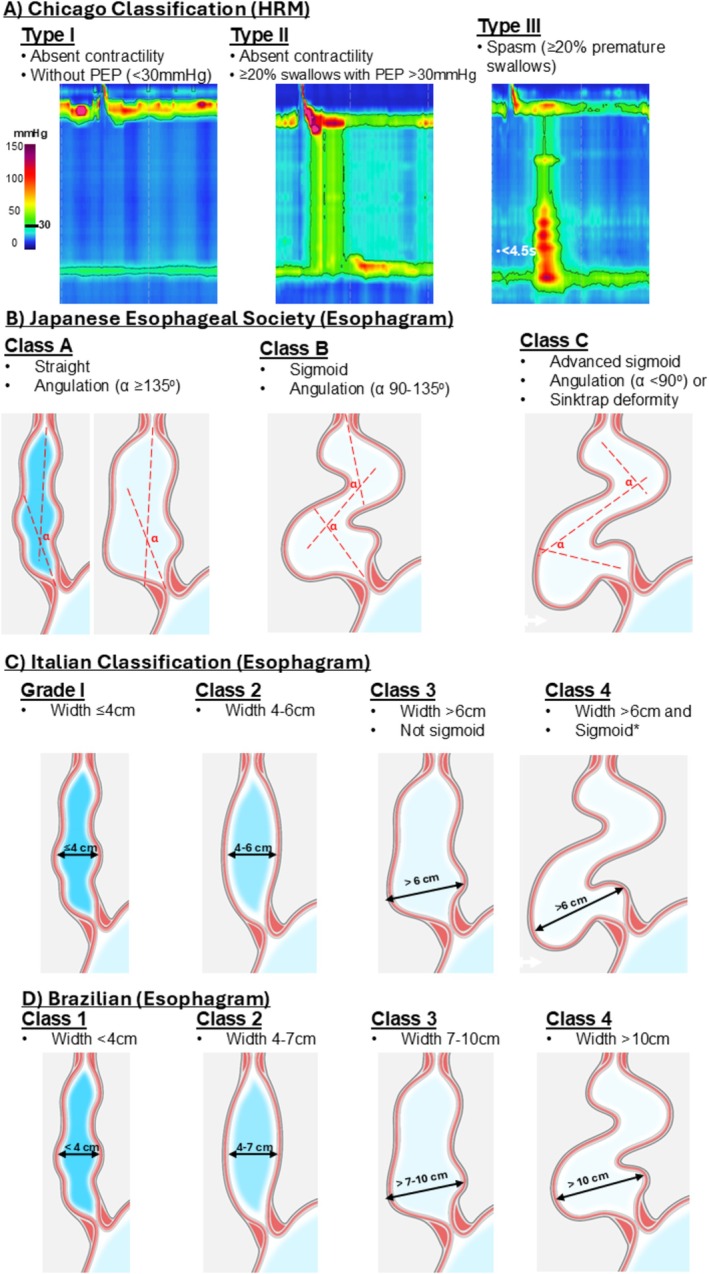
Criteria for achalasia classification schemes. High‐resolution manometry (HRM) criteria were applied to supine test swallows and also included an elevated integrated relaxation pressure (IRP) and absent peristalsis. *or advanced sigmoid regardless of esophageal width. Figure used with permission of the Esophageal Center of Northwestern.

Qualitative impression of the esophageal body morphology was assessed as straight or sigmoid and trajectory lines drawn along the esophageal long axis were utilized to assess the degree of angulation (i.e., esophageal tortuosity) to define the JES classifications (Figure 1; [13]). The maximum esophageal body width was also measured at the greatest transverse dimension on review of all available esophagram images; these were applied to define the Italian and Brazilian classifications (Figure 1; [12]).

For TBEs, the height of the barium column was measured vertically from the EGJ to the top of the barium column on the 1‐, 2‐, and 5‐min images. When TBE was completed at follow‐up, a 5‐min column height < 5 cm was considered a “good” radiographic outcome versus ≥ 5 cm considered a “poor” radiographic outcome [19].

### 2.4 Symptomatic Outcomes

Most subjects completed the Eckardt score at treatment follow‐up which was used as a measure of symptomatic treatment outcome. The Eckardt score includes four 4‐point Likert scale questions (scored 0–3) that assess the frequency of dysphagia, chest pain, and regurgitation and the degree of weight loss, with all four items summed to yield a total score of 0–12. When the Eckardt score was completed at follow‐up, a score ≤ 3 was considered a “good” symptomatic outcome versus > 3 was considered a “poor” symptomatic outcome [5, 9, 10].

### 2.5 Statistical Analysis

Results were reported as mean (SD) or median (interquartile range) depending on the data distribution. Groups were compared with the Fisher's exact test for categorical variables and ANOVA/*t*‐tests or Kruskal–Wallis/Mann–Whitney *U* for continuous variables, depending on the data distribution. Generalized linear models (GLM) with logit link functions were used to predict good symptomatic or radiographic treatment outcomes defined by post‐treatment follow‐up Eckardt score (≤ 3) or TBE (5 min column height ≤ 5 cm), respectively.

Separate GLM models were fit for each classification: one that included only the classification (unadjusted model) and one also adjusted for key confounders including age, sex, time to follow‐up, and treatment modality. To control for the potential impact of treatment type, this approach (unadjusted and adjusted models) was then also applied only for patients treated with POEM (POEM subgroup analysis) as this was the most common treatment in the cohort and has become the most common treatment choice for patients with achalasia. Variation in outcomes was evaluated across groups within models (e.g., across JES classes) using likelihood ratio tests for adjusted models and Fisher's exact test for unadjusted models (*α* = 0.05). Noting the overlap in case classifications between the different schemes, comparisons across classifications used the Akaike information criteria (AIC) as this model provides prediction error of each of the classification schemes independent of each other, which differs from other regression models that could be subject to multicollinearity when correlated variables (such as these classification schemes) are utilized within the same model. AIC is a standard estimator of prediction error for statistical models that has long been used as a metric to compare models and has been shown to be equivalent to model selection based on cross‐validation error under broad conditions [20, 21]. Within‐sample accuracy and area under the receiver operating characteristic curve (AUROC) for each model. Because AIC quantifies prediction error (also referred to as the relative information loss) of models, lower AIC values are interpreted as improved model fit [22]. Missing outcome data (i.e., post‐treatment Eckardt score or post‐treatment TBE) was addressed using multiple imputation with chained equations (*m* = 80 imputations). Unless otherwise specified, a two‐tailed *p* value < 0.05 was considered to meet statistical significance.

## 3 Results

### 3.1 Subjects

222 patients, mean (SD) age 56 (16), 110 (49%) female, patients with achalasia were included (Table 1; Figure S1). There were 144 patients (65%) treated with POEM (POEM subgroup), while 42 (19%) were treated with PD and 36 (16%) were treated with LHM. Treatment type did not significantly differ between achalasia classifications, including Chicago (*p* = 0.061), JES (*p* = 0.692), Italian (*p* = 0.113), or Brazilian (*p* = 0.278) classification. However, numerically, pneumatic dilation was less frequent and POEM more frequent for type III achalasia (Table S1). Among Type III achalasia patients, 20/33 (61%) POEMs were performed with extended (> 7 cm) myotomy, while only 1 type III achalasia patient was treated with pneumatic dilation.

**TABLE 1:** Cohort characteristics.

Characteristic	Total cohort	POEM subgroup
*N*/*n*	222	144
Age, mean (SD), years	53 (16)	54 (17)
Sex, female, *n* (%)	110 (50)	65 (45)
On opioids, *n*/*n* ^a^ (%)	13/185 (7)	11/118 (9)
Chicago Classification (HRM), *n* (%)		
I	72 (34)	42 (29)
II	117 (52)	77 (54)
III	33 (15)	25 (17)
JES Classification (esophagram), *n* (%)		
A	158 (71)	103 (71)
B	48 (21)	31 (22)
C	16 (7)	10 (7)
Italian Classification (esophagram), *n* (%)		
I	108 (49)	78 (54)
II	78 (35)	46 (32)
III	20 (9)	10 (7)
IV	16 (7)	10 (7)
Brazilian Classification (esophagram), *n* (%)		
1	109 (49)	78 (54)
2	101 (46)	60 (42)
3	12 (5)	6 (4)
4	0	0
Treatment modality, *n* (%)		
POEM	144 (65)	144 (100)
Pneumatic dilation	42 (19)	0
LHM	36 (16)	0
Available outcome data, *n* (%)		
TBE	200 (90)	128 (89)
Eckardt score	204 (92)	136 (94)

Abbreviations: JES, Japanese Esophageal Society; LHM, Laparoscopic Heller's Myotomy; POEM, PerOral Endoscopic Myotomy; TBE, timed barium esophagram.

^a^ Data available.

Esophagram classifications differed by HRM/Chicago Classification subtypes with type I achalasia having the lowest proportion of JES‐A and Brazilian‐1 and greatest proportions of JES‐C, Italian‐I, and Brazilian‐3 (Table S1). The relationships between JES, Italian, and Brazilian classifications are illustrated in Table S2.

For the total cohort and POEM subgroup, post‐treatment symptomatic outcome with Eckardt score was completed in 204 (92%) and 136 (94%) patients at a median (IQR) 9.5 (6.5–15.5) months and 8.4 (6.2–14.2) months after treatment, respectively (Figure S1). Post‐treatment radiographic outcome with TBE was completed in 200 (90%) and 128 (89%) patients at a median (IQR) 9.7 (6.7–15.2) and 8.3 (6.3–14.2) months after treatment, respectively. 186 (84%) patients and 123 (85%), respectively, completed both TBE and Eckardt score: 93 (50%) had good Eckardt score and good TBE outcome, 27 (15%) had poor Eckardt score and poor TBE outcome; 36 (19%) had good Eckardt score outcome, but poor TBE outcome and 30 (16%) had poor Eckardt score outcome, but good TBE outcome.

### 3.2 Associations of Achalasia Classifications With Symptomatic Outcome

**FIGURE 2:**
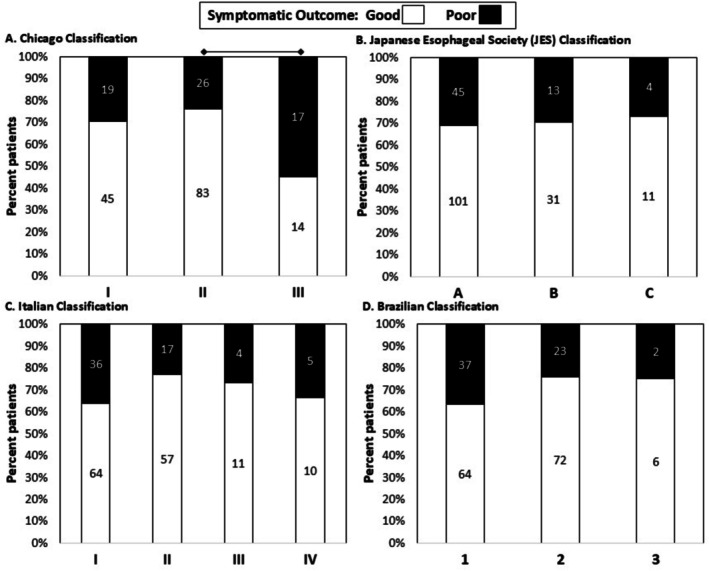
Symptomatic outcomes among achalasia classification schemes. Data labels indicate number of patients for (A) Chicago Classification, (B) Japanese Esophageal Society classification, (C) Italian classification, and (D) Brazilian classification. Lines above the bar graphs indicate significant pairwise comparisons (i.e., *p* < 0.05 after applying Bonferroni correction).

In unadjusted models, the Chicago Classification (*p* = 0.007) had a statistically significant association with symptomatic outcomes, while the other classifications did not (JES *p* = 0.940; Italian *p* = 0.283; Brazilian *p* = 0.117); Figure 2. For the Chicago Classification, there were fewer type III achalasia with good symptomatic outcomes than type II achalasia, without a significant difference between subtypes I and II. Similar results were observed when examining the Eckardt score as a continuous variable (Figure S2) and in the POEM subgroup.

Similar in models that adjusted for confounders, the Chicago Classification (*p* = 0.003) was a statistically significant predictor of symptomatic outcome, but JES (*p* = 0.967), Italian (*p* = 0.192), or Brazilian (*p* = 0.085) were not (Table 2). Across the adjusted models, the Chicago Classification exhibited the lowest AIC (258.5), suggesting the strongest predictive power relative to the other schemes (listed in order of AIC): Brazilian (265.1), Italian (268.7), JES (271.2) (Table 2). AUROCs were similar between the four schemes (0.68–0.73). The Chicago Classification also had the lowest AIC relative to the other classification schemes in the unadjusted model (Table S3), while the Brazilian Classification had the lowest AIC in the POEM subgroup model (Table S4).

**TABLE 2:** Summary of adjusted model results for prediction of symptomatic and radiographic outcomes in achalasia.

Scheme	Symptomatic outcome			Radiographic outcome		
	AIC	AUROC	LR Test	AIC	AUROC	LR Test
		(95% CI)	*p*		(95% CI)	*p*
Chicago Classification	258.5	0.72 (0.65–0.80)	0.003	283.3	0.72 (0.65–0.80)	0.098
JES	271.2	0.68 (0.60–0.76)	0.967	271.9	0.75 (0.67–0.82)	0.003
Italian	268.7	0.73 (0.65–0.81)	0.192	275.2	0.73 (0.65–0.81)	0.007
Brazilian	265.1	0.73 (0.66–0.81)	0.085	280.1	0.74 (0.66–0.81)	0.015

*Note:* Values reflect Akaike information criteria (AIC) or the within‐sample areas under the receiver operating characteristic curve (AUROC), respectively.

Abbreviations: CI, confidence interval; JES, Japaneses Esophageal Society.

### 3.3 Associations of Achalasia Classifications With Radiographic Outcome

**FIGURE 3:**
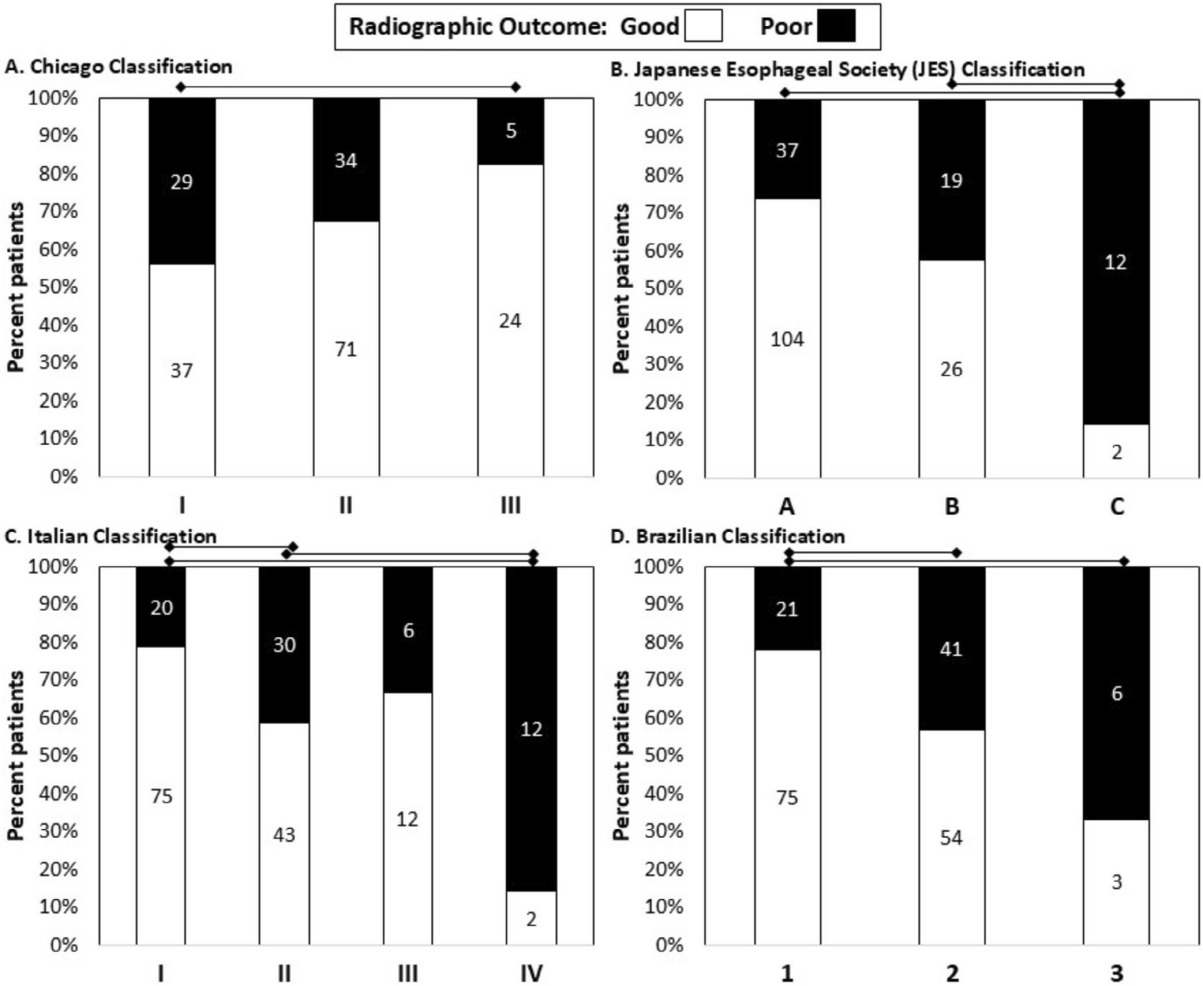
Radiographic outcomes among achalasia classification schemes. Data labels indicate number of patients for (A) Chicago Classification, (B) Japanese Esophageal Society classification, (C) Italian classification, (D) Brazilian classification. Lines above the bar graphs indicate significant pairwise comparisons (i.e., *p* < 0.05 after applying Bonferroni correction).

In unadjusted models, the Chicago (*p* = 0.031), JES (*p* < 0.001), Italian (*p* < 0.001), and Brazilian (*p* = 0.002) classifications all exhibited statistically significant associations with radiographic outcomes (Figure 3). Similar results were observed when examining the 1, 2, and 5 min column heights as continuous variables, except for not observing a significant difference with 1 min column height between the Brazilian classes (*p* = 0.069) (Figure S3). For the Chicago Classification, there were fewer type I achalasia with good radiographic outcomes than type III achalasia, without significant differences between subtypes I and II. For JES, class C had fewer good radiographic outcomes than classes A and B. In the Italian classification, grade I had fewer good radiographic outcomes than grade II or IV and grade II had fewer good outcomes than grade IV (differences were not observed between grade III and other classes). In the Brazilian Classification, class 3 and class 2 had fewer good radiographic outcomes than class 1.

In unadjusted models on the POEM subgroup, the Chicago (*p* = 0.017), JES (*p* < 0.001), Italian (*p* = 0.010), and Brazilian (*p* = 0.039) remained statistically significant predictors of radiographic outcomes. Significant differences were also observed for the 1, 2, and 5 min column heights as continuous variables for the Chicago, JES, and Italian schemes, though significant differences were not observed in the 1, 2, or 5 min column heights for the Brazilian Classification (*p*‐values 0.153, 0.080, and 0.076 respectively).

In adjusted models, JES (*p* = 0.003), Italian (*p* = 0.007), and Brazilian (*p* = 0.015) Classifications were statistically significant predictors of radiographic outcomes, but Chicago Classification (*p* = 0.098) was not. Across the adjusted models, the JES classification exhibited the lowest AIC (271.9), followed by Italian (275.2), Brazilian (280.1), and then Chicago Classification (283.3) (Table 2). AUROCs were similar between the four classification schemes (0.72–0.75). The Italian classification had the lowest AIC relative to the other classification schemes in the unadjusted model (Table S3). In adjusted model on the POEM subgroup, JES (*p* = 0.006) and Chicago (0.048) were statistically significant predictors of radiographic outcomes, while the Italian (*p* = 0.083) and Brazillian (*p* = 0.164) were not (Table S4). The JES classification had the lowest AIC in the POEM subgroup model (Table S4).

## 4 Discussion

The major findings of this study were that each of the four described achalasia classification schemes—the Chicago Classification based on HRM and the JES, Italian, or Brazilian schemes based on esophagram—were capable of predicting treatment outcomes in achalasia. HRM (Chicago Classification) performed better than esophagram in predicting symptomatic outcomes, while the esophagram classifications performed better in predicting objective radiographic outcomes. Hence, there appears to be potential value in utilizing both HRM and esophagram prior to achalasia treatment to aid prognostication of treatment response.

Similar to the initial achalasia HRM subtypes cohort description and subsequent follow‐up studies, we found achalasia treatment outcomes were predicted by the Chicago Classification subtypes in this cohort [1]. However, despite being treated primarily with tailored POEM (or with LHM in the remainder), type III achalasia still had the lowest rates of good symptomatic outcomes. This finding somewhat differed from the conclusions of the European Achalasia trial (randomized trial of pneumatic dilation vs. LHM in achalasia) that showed similar outcomes for type III achalasia to types I and II treated with LHM, but worse outcomes in type III treated with PD [5]. The discrepancy may reflect heterogeneity within type III achalasia and suggests that improvement in phenotyping or treating spastic achalasia remains an area in need of future investigation.

The outcome prediction with the specific JES, Italian, or Brazilian esophagram classifications is less thoroughly described than the Chicago Classification. The present study provided an external, independent evaluation of each and also appears to be the first to compare these four three schemes (Chicago, JES, Italian, and Brazilian) [1, 3, 4, 5, 6, 7, 14]. Similar to a study of 59 achalasia patients treated with LHM, symptomatic treatment outcomes did not differ between JES stages [14]. The previous study utilizing the Italian classification, which included 1001 patients treated with LHM, had demonstrated a significant difference in symptomatic outcome between the Italian esophagram grades. This in particular was related to sigmoid esophagus (grade IV), which was also associated with higher rates of treatment failure in other studies [6, 7]. Accompanying the Brazilian classification, a pooled analysis of case series of treatment outcomes for end‐stage achalasia suggested that advanced stages can often be successfully treated with LHM (i.e., similar outcomes to early stages), thus also showing similar results with symptomatic outcomes in this study [12]. The current study may be the largest study specifically describing treatment outcomes with the Brazilian classification scheme, noting however that there were no patients with Brazilian stage 4 included in this cohort.

While each classification uses esophagram, the JES classification is primarily driven by anatomic deformity (i.e., angulation), while the Brazilian solely by dilatation (width). The Italian classification is primarily driven by dilatation but also incorporates deformity (sigmoidization) into its advanced grade IV. Overall, there is variable overlap between the esophagram‐based schemes, as well as the esophagram schemes with HRM/Chicago Classification, which suggests the potential benefit of using both modalities to phenotype patients with achalasia. Seeking to optimize the approaches to phenotype achalasia with the potential to prognosticate and direct management strategies is an ongoing effort.

A strength of this study is the incorporation of an objective outcome of esophageal emptying using a standardized TBE protocol beyond solely relying on subjective outcome measures. In doing so, it was notable that type III achalasia by the Chicago Classification had higher rates of good radiographic outcomes, despite having the lowest rates of good symptomatic outcomes, compared with subtypes I and II. Further, advanced JES (C), Italian (IV), and Brazilian (2–3) classifications had higher rates of poor radiographic outcomes (compared with classes A‐B, I‐II, or 1, respectively), despite a lack of difference in symptomatic outcomes. Discordance between symptoms and objective esophageal retention is a phenomenon in achalasia previously described in seminal studies from Vaezi et al. [23, 24, 25] These prior studies demonstrated that radiographic retention was a key predictor for the need for retreatment in achalasia (i.e., achalasia treatment failure), and a stronger predictor than symptoms [24]. This supports the importance of using retention on TBE as an outcome to assess treatment response in achalasia. This also highlights that interactions between motility (i.e., failed vs. spastic contractility), pressurization, and anatomy, as well as other factors such as hypervigilance or anxiety, can impact outcomes in achalasia [26]. Further, this suggests that the relative weights of these factors on outcomes may vary across stages of the achalasia disease course, which also represents an anticipated focus for future investigation.

While this study offers strengths related to its comprehensive analysis, there are limitations. Both the subjective (Eckardt score) and objective (TBE) treatment outcome measures carry inherent limitations in isolation, as does dichotomizing outcomes. The observational nature of the study also carries inherent limitations, including that treatment choices were not standardized. In particular, the Chicago Classification was directly applied clinically for treatment planning (i.e., type III achalasia were selectively treated with POEM or LHM), whereas the radiographic schemes were not, which could potentially dampen the prognostic benefits of the Chicago Classification relative to the radiographic schemes. However, we attempted to address treatment effects using the modeling approaches to adjust for potential confounders, including treatment type (i.e., the adjusted analysis among the total cohort), as well as assessing the focused POEM subgroup. Further, as this study included newly diagnosed achalasia (i.e., without previous treatment), radiographic classes with advanced deformity were relatively infrequent (including zero Brazilian class 4), which also may have impacted results. Additionally, patient loss to follow‐up which limited available outcome data may have generated a selection bias, potentially overrepresenting poor outcomes, for example, if symptoms or retention prompted patients to return for evaluation. Finally, mechanisms of treatment failure in achalasia are variable and not all are likely capable of being predicted based on baseline data. Future study will be enhanced by additional characterization of treatment failures relative to baseline classification.

In conclusion, the present study demonstrated the performance of achalasia classification schemes to prognosticate treatment outcomes. While treatment choice may impact outcomes, HRM (Chicago Classification) performed better than esophagram to predict symptomatic outcomes, while the esophagram classifications performed better to predict objective radiographic outcomes. Anticipated future directions include development of a multi‐modal scheme that leverages the collective benefits from each to further help phenotype patients with achalasia. Ultimately, these efforts seek to improve our approaches to diagnosis and manage achalasia.

## Author Contributions

D.A.C. contributed to study concept and design, obtaining funding, drafting of the manuscript, data analysis, data interpretation, and approval of the final version. E.G., D.A.F., L.C., and L.K. contributed to data analysis, data interpretation, and approval of the final version. J.M.S. contributed to data analysis, data interpretation, editing the manuscript critically, and approval of the final version. J.E.P. contributed to study concept and design, obtaining funding, data interpretation, editing the manuscript critically, and approval of the final version.

## Funding

This work was supported by R01 DK137775 (J.E.P. and D.A.C.) from the Public Health service (NIDDK).

## Conflicts of Interest

D.A.C.: Medtronic (speaking, consulting, license); Diversatek (consulting); Braintree (consulting); Medpace (consulting); Phathom Pharmaceuticals (Speaking; consulting); Regeneron/Sanofi (speaking; consulting); Laborie (consulting). J.E.P.: Sandhill Scientific/Diversatek (consulting, grant), Takeda (speaking), Astra Zeneca (Speaking), Medtronic (speaking, consulting, patent, license), Torax/Ethicon (speaking, consulting), EndoGastric Solutions (Advisory Board), Phathom (speaking, consulting); Laborie (consulting). Other authors have no conflicts to disclose.

## Supporting information


**Table S1:** Characteristics by Chicago Classification achalasia subtypes. *p < 0.05 on comparison between High‐resolution manometry (HRM)/Chicago Classification achalasia subtypes. ^a^Data available. JES, Japanese Esophageal Society; LHM, Laparoscopic Heller's Myotomy; POEM, PerOral Endoscopic Myotomy; TBE, timed barium esophagram.
**Table S2:** Relationships of esophagram achalasia classifications. Values represent number of patients.
**Table S3:** Summary of unadjusted model results for prediction of symptomatic and radiographic outcomes in achalasia. Values reflect Akaike information criteria (AIC) or the within‐sample areas under the receiver operating characteristic curve (AUROC), respectively. CI, confidence interval; JES, Japaneses Esophageal Society.
**Table S4:** Summary of adjusted model results for prediction of symptomatic and radiographic outcomes in the sub‐group of achalasia patients treated with POEM (POEM subgroup model). Values reflect Akaike information criteria (AIC) or the within‐sample areas under the receiver operating characteristic curve (AUROC), respectively. CI, confidence interval; JES, Japanese Esophageal Society; LR, Likelihood ratio.
**Figure S1:** Patient flow. HRM, high‐resolution manometry; LHM, laparoscopic Heller's myotomy; POEM, PerOral Endoscopic Myotomy; TBE, timed barium esophagram.
**Figure S2:** Symptomatic outcomes with Eckardt score as a continuous variable among achalasia classification schemes. (A) Chicago Classification, (B) Japanese Esophageal Society stages, (C) Italian Classification, and (D) Brazilian Classification. Horizontal dashed lines were placed between scores 3 and 4 in each panel. “0” and “*” indicate outlier values.
**Figure S3:**. Radiographic outcomes as timed barium esophagram column heights among achalasia classification schemes. (A) Chicago Classification, (B) Japanese Esophageal Society stages, (C) Italian classification, and (D) Brazilian classification. Horizontal dashed lines were placed at the 5‐cm column height in each panel. “0” and “*” indicate outlier values.

## Data Availability

The data that support the findings of this study are available from the corresponding author upon reasonable request and completion of necessary privacy and ethical approvals.
