# Supplementary material for: Comparison of Achalasia Classification Schemes to Predict Treatment Outcomes
Source: Neurogastroenterol Motil. 2026 Jan 20;38(1):e70249. doi: 10.1111/nmo.70249 (PMC12818386; doi:10.1111/nmo.70249)

**Supplemental material**

**Table S1. Characteristics by Chicago Classification Achalasia Subtypes.** * = P<0.05 on comparison between High-resolution manometry (HRM)/Chicago Classification achalasia subtypes. ^a^ Data available. JES – Japanese Esophageal Society. POEM – PerOral Endoscopic Myotomy. LHM – Laparoscopic Heller’s Myotomy. TBE – timed barium esophagram

|  | **HRM-Chicago Classification Achalasia subtypes** | | |
| --- | --- | --- | --- |
|  | **Type I achalasia** | **Type II achalasia** | **Type III achalasia** |
| **N / n** | 72 | 117 | 33 |
| **Age, mean (SD), years*** | 54 (15) | 51 (17) | 61 (15) |
| **Sex, female, n (%)** | 39 (54) | 57 (49) | 14 (42) |
| **On opioids, n/n^a^ (%)*** | 1/61 (2) | 2/95 (2) | 10/29 (35) |
| **JES Classification (esophagram), n (%)**  **A**  **B**  **C** | 38 (52)  20 (28)  14 (19) | 94 (80)  21 (18)  2 (2) | 26 (79)  7 (21)  0 |
| **Italian Classification (esophagram), n (%)**  **I**  **II**  **III**  **IV** | 16 (22)  30 (42)  12 (17)  14 (19) | 61 (52)  46 (39)  8(7)  2 (2) | 31 (94)  2 (6)  0  0 |
| **Brazilian Classification (esophagram), n (%)**  **1**  **2**  **3**  **4** | 17 (24)  45 (63)  10 (14)  0 | 61 (52)  54 (46)  2 (2)  0 | 31 (94)  2 (6)  0  0 |
| **Treatment modality, n (%)**  **POEM**  **Pneumatic dilation**  **LHM** | 42 (58)  18 (25)  12 (17) | 77 (66)  23 (20)  17 (15) | 25 (76)  1 (3)  7 (21) |
| **Available outcome data, n (%)**  **TBE**  **Eckardt score** | 66 (92)  64 (89) | 105 (90)  109 (93) | 29 (88)  31 (94) |

**Table S2. Relationships of Esophagram Achalasia Classifications.** Values represent number of patients.

|  | **JES Classification** | | |  | **Italian Classification** | | | |
| --- | --- | --- | --- | --- | --- | --- | --- | --- |
|  | **A** | **B** | **C** |  | **I** | **II** | **III** | **IV** |
| **Italian Classification**  **I**  **II**  **III**  **IV** | 95  52  11  0 | 13  26  9  1 | 0  0  0  15 |  | --  --  --  -- | --  --  --  -- | --  --  --  -- | --  --  --  -- |
| **Brazilian Classification**  **1**  **2**  **3**  **4** | 96  60  3  0 | 13  32  4  0 | 1  9  5  0 |  | 108  0  0  0 | 0  78  0  0 | 0  14  6  0 | 1  9  6  0 |

**Table S3. Summary of unadjusted model results for prediction of symptomatic and radiographic outcomes in achalasia.**

Values reflect Akaike information criteria (AIC) or the within-sample areas under the receiver operating characteristic curve (AUROC), respectively. CI - confidence interval. JES-Japaneses Esophageal Society.

| **Scheme** | **Symptomatic Outcome** | | | **Radiographic Outcome** | | |
| --- | --- | --- | --- | --- | --- | --- |
|  | **AIC** | **AUROC**  **(95% CI)** | **Fisher Test**  **p-value** | **AIC** | **AUROC**  **(95% CI)** | **Fisher Test**  **p-value** |
| **Chicago Classification** | 270.4 | 0.61  (0.53-0.69) | 0.007 | 283.9 | 0.60  (0.52-0.68) | 0.031 |
| **JES** | 279.5 | 0.51  (0.42-0.60) | 0.940 | 271.6 | 0.64  (0.56-0.72) | <0.001 |
| **Italian** | 278.7 | 0.57  (0.49-0.66) | 0.283 | 270.5 | 0.68  (0.61-0.75) | <0.001 |
| **Brazilian** | 275.7 | 0.58  (0.49-0.66) | 0.117 | 278.3 | 0.64  (0.56-0.72) | 0.002 |

**Table S4. Summary of adjusted model results for prediction of symptomatic and radiographic outcomes in the sub-group of achalasia patients treated with POEM (POEM subgroup model).** Values reflect Akaike information criteria (AIC) or the within-sample areas under the receiver operating characteristic curve (AUROC), respectively. CI - confidence interval. JES-Japanese Esophageal Society. LR – Likelihood Ratio.

| **Scheme** | **Symptomatic Outcome** | | | **Radiographic Outcome** | | |
| --- | --- | --- | --- | --- | --- | --- |
|  | **AIC** | **AUROC**  **(95% CI)** | **LR Test**  **p-value** | **AIC** | **AUROC**  **(95% CI)** | **LR Test**  **p-value** |
| **Chicago Classification** | 157.5 | 0.79  (0.70-0.87) | 0.034 | 184.4 | 0.70  (0.60-0.80) | 0.048 |
| **JES** | 163.8 | 0.77  (0.68-0.86) | 0.841 | 179.2 | 0.75  (0.66-0.84) | 0.006 |
| **Italian** | 159.9 | 0.81  (0.73-0.89) | 0.162 | 184.8 | 0.65  (0.54-0.76) | 0.083 |
| **Brazilian** | 156.7 | 0.80  (0.71-0.88) | 0.046 | 187.8 | 0.65  (0.55-0.76) | 0.164 |

**Supplemental Figures:**

**Figure S1. Patient flow.** HRM – high-resolution manometry; POEM – PerOral Endoscopic Myotomy; LHM – laparoscopic Heller’s myotomy; TBE – timed barium esophagram

**
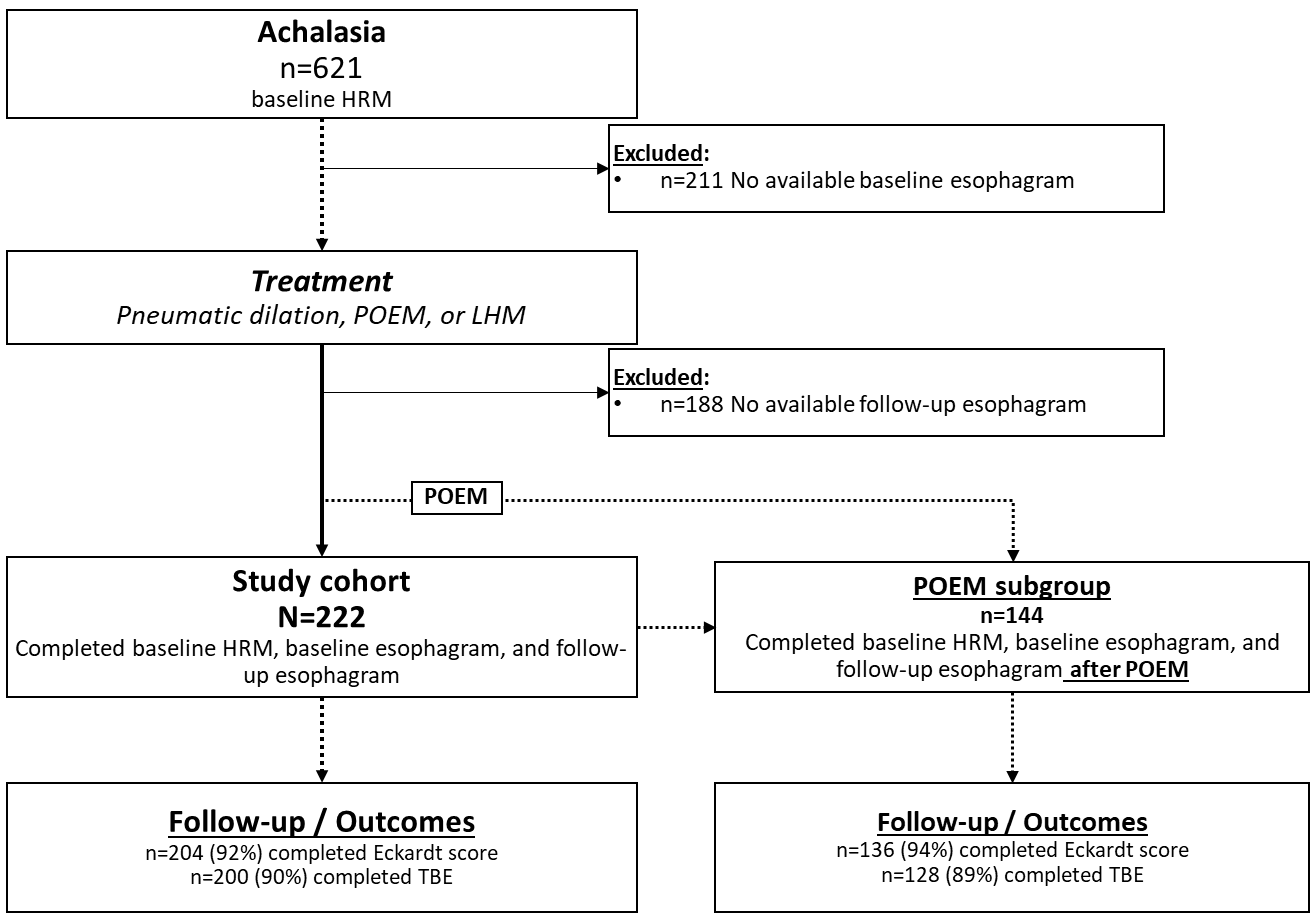
**

**Figure S2. Symptomatic outcomes with Eckardt score as a continuous variable among achalasia classification schemes.** A) Chicago Classification, B) Japanese Esophageal Society stages, C) Italian Classification, and D) Brazilian Classification. Horizontal dashed lines were placed between scores 3 and 4 in each panel. “⁰” and “*” indicate outlier values.


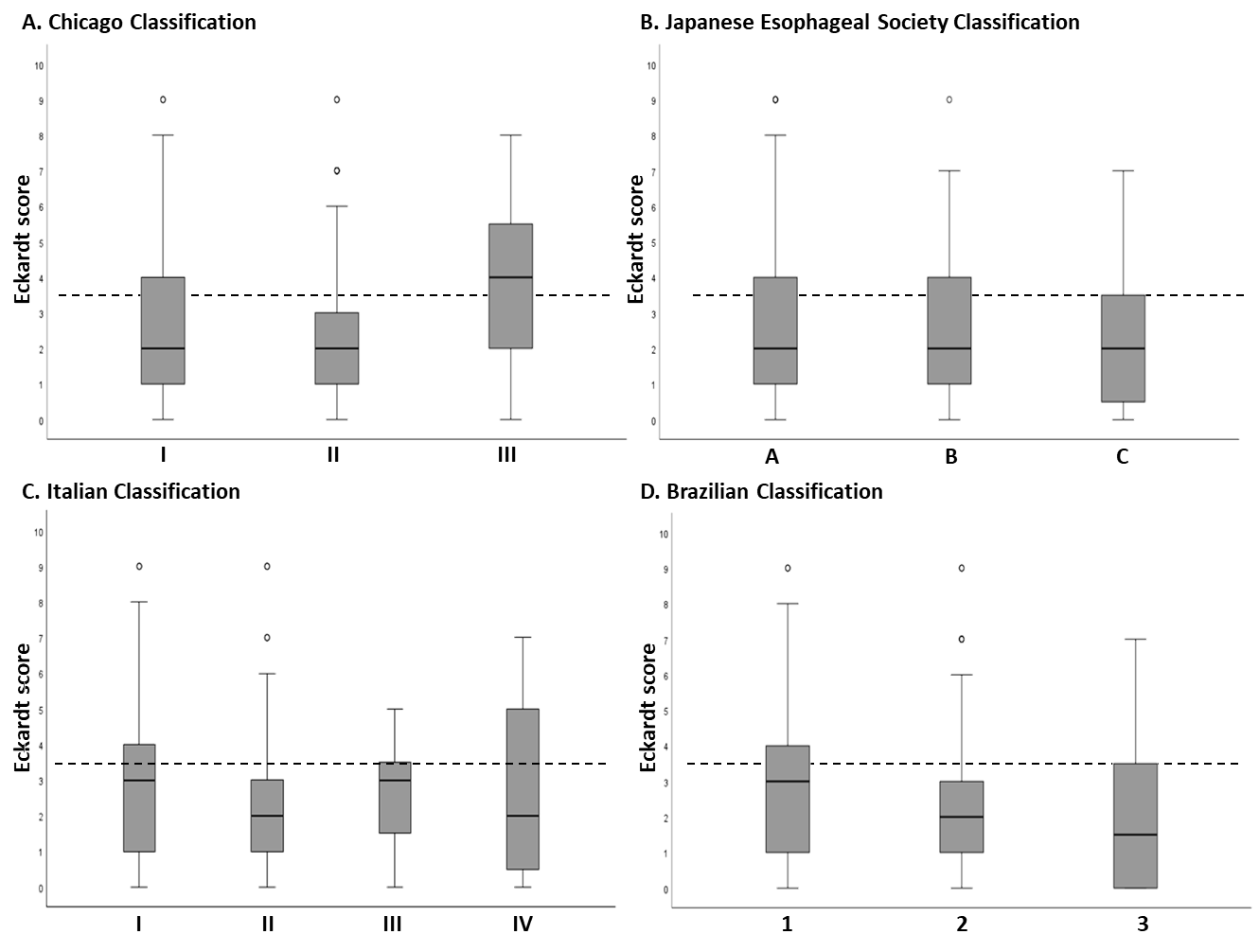


**Figure S3. Radiographic outcomes as timed barium esophagram column heights among achalasia classification schemes.** A) Chicago Classification, B) Japanese Esophageal Society stages, C) Italian Classification, and D) Brazilian Classification. Horizontal dashed lines were placed at the 5-cm column height in each panel. “⁰” and “*” indicate outlier values.


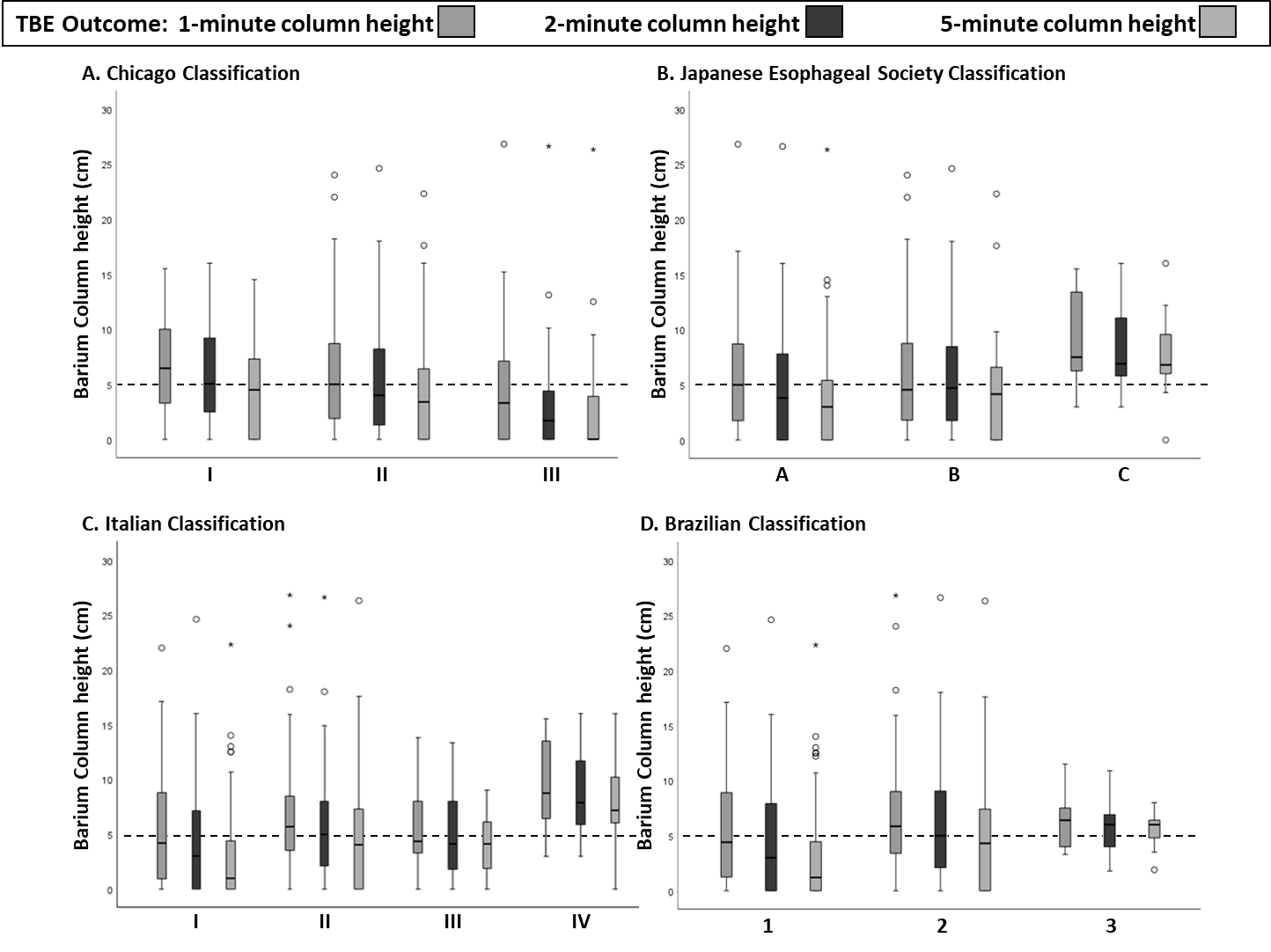

Supplement: Supplementary file 1 — Table S1: Characteristics by Chicago Classification achalasia subtypes. *p < 0.05 on comparison between High‐resolution manometry (HRM)/Chicago Classification achalasia subtypes. aData available. JES, Japanese Esophageal Society; LHM, Laparoscopic Heller's Myotomy; POEM, PerOral Endoscopic Myotomy; TBE, timed barium esophagram. Table S2: Relationships of esophagram achalasia classifications. Values represent number of patients. Table S3: Summary of unadjusted model results for prediction of symptomatic and radiographic outcomes in achalasia. Values reflect Akaike information criteria (AIC) or the within‐sample areas under the receiver operating characteristic curve (AUROC), respectively. CI, confidence interval; JES, Japaneses Esophageal Society. Table S4: Summary of adjusted model results for prediction of symptomatic and radiographic outcomes in the sub‐group of achalasia patients treated with POEM (POEM subgroup model). Values reflect Akaike information criteria (AIC) or the within‐sample areas under the receiver operating characteristic curve (AUROC), respectively. CI, confidence interval; JES, Japanese Esophageal Society; LR, Likelihood ratio. Figure S1: Patient flow. HRM, high‐resolution manometry; LHM, laparoscopic Heller's myotomy; POEM, PerOral Endoscopic Myotomy; TBE, timed barium esophagram. Figure S2: Symptomatic outcomes with Eckardt score as a continuous variable among achalasia classification schemes. (A) Chicago Classification, (B) Japanese Esophageal Society stages, (C) Italian Classification, and (D) Brazilian Classification. Horizontal dashed lines were placed between scores 3 and 4 in each panel. “0” and “*” indicate outlier values. Figure S3:. Radiographic outcomes as timed barium esophagram column heights among achalasia classification schemes. (A) Chicago Classification, (B) Japanese Esophageal Society stages, (C) Italian classification, and (D) Brazilian classification. Horizontal dashed lines were placed at the 5‐cm column height in each pan [file NMO-38-e70249-s001.zip › Achalasia Classification comparison_supplemental.docx]
